# Stem Cell-Derived Exosomes Ameliorate Doxorubicin-Induced Muscle Toxicity through Counteracting Pyroptosis

**DOI:** 10.3390/ph13120450

**Published:** 2020-12-09

**Authors:** Fatima Bianca A. Dessouki, Rakesh C. Kukreja, Dinender K. Singla

**Affiliations:** 1 Division of Metabolic and Cardiovascular Sciences, Burnett School of Biomedical Sciences, College of Medicine, University of Central Florida, Orlando, FL 32816, USA; biancadessouki@knights.ucf.edu; 2 Pauley Heart Center, Virginia Commonwealth University, Richmond, VA 23298, USA; rakesh.kukreja@vcuhealth.org

**Keywords:** atrophy, embryonic stem cells, exosomes, inflammation, macrophage, muscle toxicity

## Abstract

Doxorubicin (Dox)-induced muscle toxicity (DIMT) is a common occurrence in cancer patients; however, the cause of its development and progression is not established. We tested whether inflammation-triggered cell death, “pyroptosis” plays a role in DIMT. We also examined the potential role of exosomes derived from embryonic stem cells (ES-Exos) in attenuating DIMT. C57BL/6J mice (10 ± 2 wks age) underwent the following treatments: Control (saline), Dox, Dox+ES-Exos, and Dox+MEF-Exos (mouse-embryonic fibroblast-derived exosomes, negative control). Our results demonstrated that Dox significantly reduced muscle function in mice, which was associated with a significant increase in NLRP3 inflammasome and initiation marker TLR4 as compared with controls. Pyroptosis activator, ASC, was significantly increased compared to controls with an upregulation of specific markers (caspase-1, IL-1β, and IL-18). Treatment with ES-Exos but not MEF-Exos showed a significant reduction in inflammasome and pyroptosis along with improved muscle function. Additionally, we detected a significant increase in pro-inflammatory cytokines (TNF-α and IL-6) and inflammatory M1 macrophages in Dox-treated animals. Treatment with ES-Exos decreased M1 macrophages and upregulated anti-inflammatory M2 macrophages. Furthermore, ES-Exos showed a significant reduction in muscular atrophy and fibrosis. In conclusion, these results suggest that DIMT is mediated through inflammation and pyroptosis, which is attenuated following treatment with ES-Exos.

## 1. Introduction

Doxorubicin (Dox) is an effective chemotherapeutic drug that is used to treat a wide variety of cancers such as breast, lung, leukemia, and lymphoma through inhibition of topoisomerase II, DNA intercalation, and oxidative stress [1,2,3,4]. Yet, the use of Dox is restricted due to dose-dependent, severe, acute cardiac toxicity and related reversible side effects such as arrhythmia and hair loss [5,6,7,8,9,10]. Clinical data and studies from our and other laboratories have established that Dox chemotherapy also induces muscle toxicity (DIMT) [5,11,12,13,14,15]. Toxicity in skeletal muscle tissue can often lead to impairment of quality of life and in some severe cases, can be lethal [16]. Symptoms such as muscle weakness, myalgia, or myoglobinuria are associated with drug-induced muscle toxicity [16]. This is a significant health issue in cancer patients treated with Dox and currently there is little information about the specific causes of DIMT. Various cellular and pathophysiological changes in the soleus muscle are implicated in DIMT such as oxidative stress, fibrosis, atrophy, and apoptotic cell death [11,12,17]. These changes cause an imbalance in cell death and cell survival, which severely impacts muscle tissue, causing deterioration, and ultimately muscle dysfunction. We have previously reported the role of inflammation-mediated cell death, pyroptosis, in slow-twitch soleus muscle (Sol 8) cells in DIMT [5].

Pyroptosis, an inherent mechanism of cell death associated with the inflammatory reaction found in various inflammation-associated diseases such as cardiovascular, diabetes, and cancer [5,18,19,20,21,22,23]. Pyroptosis is characterized by NLR family pyrin domain containing 3-apoptosis-associated speck-like protein containing a caspase recruitment domain (NLRP3-ASC) inflammasome formation and activation of Caspase-1, which is the distinguishing feature from other cell death mechanisms [22,24,25,26]. This contributes to membrane pore formation and maturation of pro-inflammatory cytokines IL-1β and IL-18, facilitating cell death [18,25,27]. Recently, we showed that pyroptosis played a role in Dox-induced cardiotoxicity (DIC) [21,22] although its impact in causing DIMT in vivo remains enigmatic. Moreover, the potential source of inflammation, which may play a role in the progression of Dox-induced myopathy, is poorly understood.

As stated above, currently there are no therapeutic strategies available to effectively mitigate DIMT in cancer patients. Several studies have suggested that embryonic stem (ES) cells are promising candidates for transplantation therapy, although formation of teratoma (tumorigenesis) remains one of the major limitations [28,29,30,31]. Fortunately, secreted vesicles, called exosomes (Exos), derived from ES cells offer an attractive alternative approach to cell-based therapy [5,22,32,33]. However, the in vivo effect of ES-Exos in attenuating DIMT remains unknown. In the current study we sought to investigate the role of pyroptosis, inflammation, and the potential influx of cellular source of inflammation. In addition, we investigated whether Dox-induced pyroptosis plays a role in muscular atrophy and fibrosis which may lead to muscle dysfunction. The therapeutic effects of ES-Exos were examined to understand their ability to attenuate inflammation, pyroptosis and modulate the potential source of inflammatory cells, which may attenuate atrophy, fibrosis and improve muscle function.

## 2. Results

### 2.1. ES-Exos Treatment Improves Dox-Induced Muscle Dysfunction

**Figure 1:**
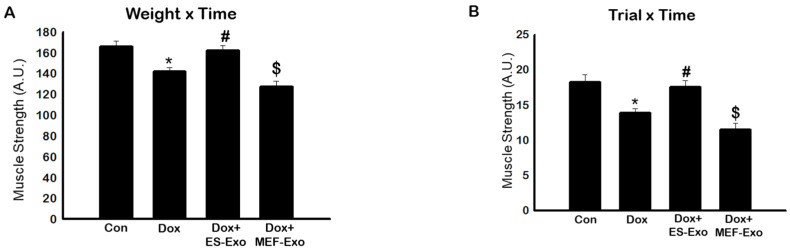
Treatment with ES-Exos improve muscle dysfunction after Dox administration. Mice were subjected to carry various weights for 3 s. If the hold was less than 3 s, the mouse was given 3 trials. (**A**) Histogram derived from scores calculated by multiplying the last weight the mouse was able to carry by the time it was able to carry the weight (weight x time, WT). (**B**) Histogram derived from scores calculated by multiplying the last trial number the mouse was able to carry a weight by the time it was able to carry the weight (trial x time, TT). Error bars = mean ± S.E.M. * *p* < 0.05 vs. control, # *p* < 0.05 vs. Dox, $ *p* = non-significant vs. Dox, one-way ANOVA followed by Tukey test, quantities are represented as arbitrary units (A.U.). n = 14–16, male and female.

The effects of Dox on muscle strength was evaluated through muscle function, calculated using weight x time method as reported previously [34]. A significant decrease in muscle function was observed by measuring weight x time (Figure 1A) in Dox-treated mice compared to the controls (*p* < 0.05). 

To further validate this data we also measured trial x time to evaluate muscle function. A significant (*p* < 0.05) reduction in muscle function was observed in Dox-treated animals as compared with controls (Figure 1B). Treatment with ES-Exos significantly improved muscle function as evaluated by weight x time and trial x time methods (*p* < 0.05, Figure 1A,B). Importantly, to understand the differential effect of ES-Exos, we used MEF-Exos, which contain different cellular components compared with ES-Exos. There was no significant improvement following MEF-Exos treatment, suggesting that ES-Exos have different cellular protective components, which improve muscle function.

### 2.2. ES-Exos Treatment Inhibits the Formation of the NLRP3-ASC Inflammasome after Dox Administration

**Figure 2:**
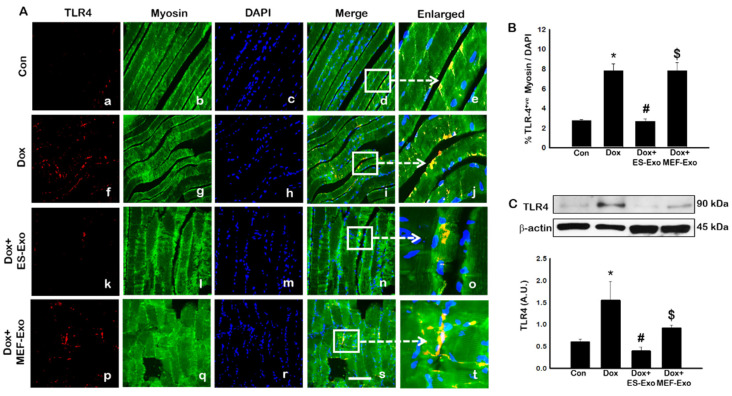
Treatment with ES-Exos inhibits TLR4 activation in soleus muscle. (**A**) Representative photomicrographs of soleus muscle sections stained with myosin and inflammasome marker TLR4. Each box shows TLR4^+^ cells in red (**a**,**f**,**k**,**p**), myocytes in green (**b**,**g**,**l**,**q**), DAPI in blue (**c**,**h**,**m**,**r**), merged images (**d**,**i**,**n**,**s**), and enlarged areas of merged images (**e**,**j**,**o**,**t**). (**B**) Histograms from quantitative analysis of TLR4^+^ cells over total DAPI. (**C**) Representative blot and densitometric analysis of TLR4. Error bars = mean ± S.E.M. * *p* < 0.05 vs. control, # *p* < 0.05 vs. Dox, $ *p* = non-significant vs. Dox, one-way ANOVA followed by Tukey test; western blot quantities are represented as A.U. Scale bar = 100 µm, n = 14–16, male and female (**B**) and n = 6–8, male and female (**C**). Images taken at 40× magnification.

**Figure 3:**
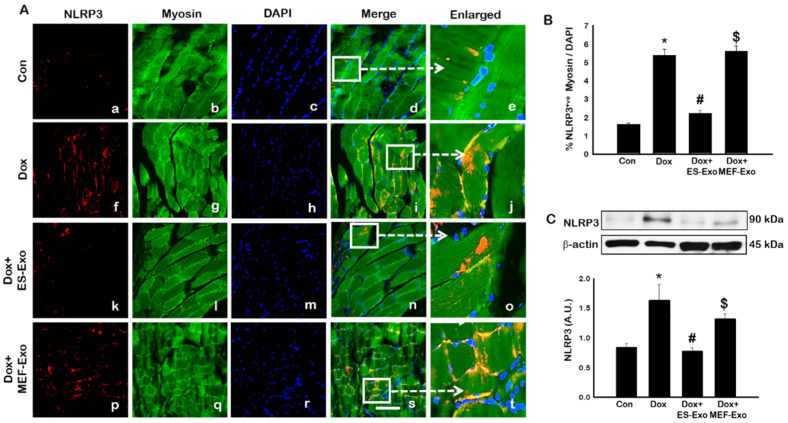
ES-Exos treatment of mice inhibits NLRP3 inflammasome formation in the soleus muscle after Dox administration. (**A**) Representative photomicrographs of soleus muscle sections stained with myosin and inflammasome marker NLRP3. Each box shows NLRP3^+^ cells in red (**a**,**f**,**k**,**p**), myocytes in green (**b**,**g**,**l**,**q**), DAPI in blue (**c**,**h**,**m**,**r**), merged images (**d**,**i**,**n**,**s**), and enlarged areas of merged images (**e**,**j**,**o**,**t**). (**B**) Histograms from quantitative analysis of NLRP3^+^ cells over total DAPI. (**C**) Representative blot and densitometric analysis of NLRP3. Error bars = mean ± S.E.M. * *p* < 0.05 vs. control, # *p* < 0.05 vs. Dox, $ *p* = non-significant vs. Dox, one-way ANOVA followed by Tukey test; western blot quantities are represented as A.U. Scale bar = 100 µm, n = 14–16, male and female (**B**) and n = 6–8, male and female (**C**). Images taken at 40× magnification.

To investigate the role of pyroptosis in Dox-induced myopathy, we first examined inflammasome formation in muscle. Immunohistochemical (IHC) analysis showed that mice treated with Dox showed increased number of TLR4^+^ cells (Figure 2A(f–j)), NLRP3^+^ cells (Figure 3A(k–o)) as compared to control (a–e).This significant increase of TLR4^+^ (Figure 2A(k–o)) and NLRP3^+^ cells (Figure 3A(k–o)) were reduced upon ES-Exos treatment, suggesting the potential impact of ES-Exos in the attenuation of the inflammasome complex formation. No such significant reduction of inflammasome^+^ markers was observed in MEF-Exos treatment (p–t). The differences in the number of positive cells with and without treatment were quantified and shown as histograms for TLR4 (Figure 2B, *p* < 0.05) and NLRP3 (Figure 3B, *p* < 0.05). To add to our IHC data, we performed western blot analysis. Our data shows that Dox-administered mice exhibited a significant increase in TLR4 (Figure 2C, *p* < 0.05) and NLRP3 (Figure 3C, *p* < 0.05) vs. control mice, whereas treatment with ES-Exos significantly lowered these protein levels compared to Dox (*p* < 0.05).

**Figure 4:**
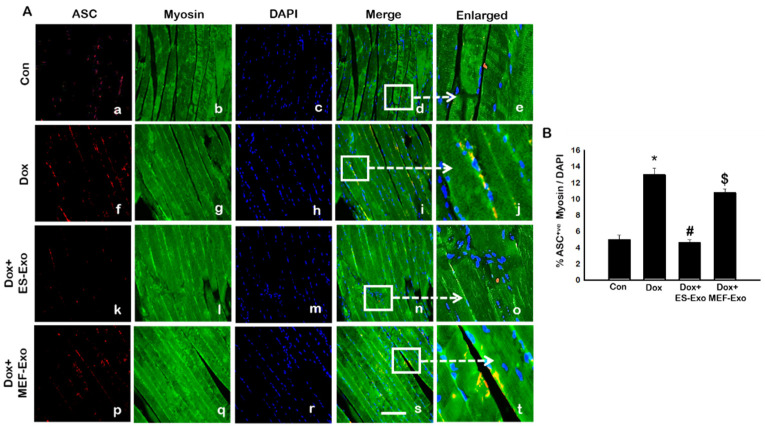
Treatment with ES-Exos inhibits adapter protein, ASC, after Dox administration. (**A**) Representative photomicrographs of soleus muscle sections stained with myosin and adapter protein, ASC. Each box shows ASC^+^ cells in red (**a**,**f**,**k**,**p**), myocytes in green (**b**,**g**,**l**,**q**), DAPI in blue (**c**,**h**,**m**,**r**), merged images (**d**,**i**,**n**,**s**), and enlarged areas of merged images (**e**,**j**,**o**,**t**). (**B**) Histograms from quantitative analysis of ASC^+^ cells over total DAPI. Error bars = mean ± S.E.M. * *p* < 0.05 vs. control, # *p* < 0.05 vs. Dox, $ *p* = non-significant vs. Dox, one-way ANOVA followed by Tukey test; Scale bar = 100 µm, n = 14–16, male and female. Images taken at 40× magnification.

We further examined whether ASC is involved in the polymerization process of the inflammasome complex, which is implicated in pyroptotic cell death [24,26,27,35,36,37]. The results show a significant increase in the number of skeletal muscle cells positive for ASC in Dox-treated groups compared with controls (Figure 4A), which is attenuated by ES-Exos treatment. 

Treatment with MEF-Exos did not show significant decrease in ASC^+^ cells as compared with Dox -treated groups (Figure 4B). Quantitative analysis shows that Dox-treated animals had 13% ASC^+^ cells compared with 5% in control animals (Figure 4B, *p* < 0.05). Treatment with ES-Exos significantly (*p* < 0.05) reduced the number of ASC^+^ cells reaching a level close to controls, whereas no statistically significant reduction in ASC^+^ cells were observed by MEF-Exos treatment.

### 2.3. ES-Exos Treatment Reduces Dox-Induced Pyroptotic Cascade

**Figure 5:**
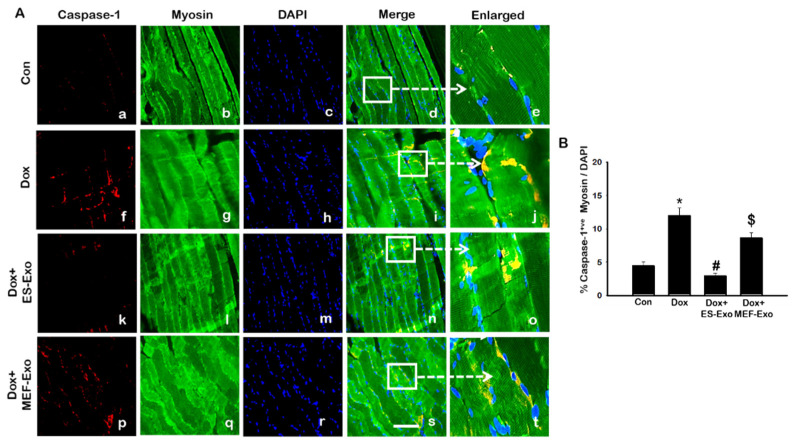
Treatment with ES-Exos reduces Dox-induced caspase-1 cascade. (**A**) Representative photomicrographs of soleus muscle sections stained with myosin and pyroptotic marker, caspase-1. Each box shows caspase-1^+^ cells in red (**a**,**f**,**k**,**p**), myocytes in green (**b**,**g**,**l**,**q**), DAPI in blue (**c**,**h**,**m**,**r**), merged images (**d**,**i**,**n**,**s**), and enlarged areas of merged images (**e**,**j**,**o**,**t**). (**B**) Histograms from quantitative analysis of caspase-1^+^ cells over total DAPI. Error bars = mean ± S.E.M. * *p* < 0.05 vs. control, # *p* < 0.05 vs. Dox, $ *p* = non-significant vs. Dox, one-way ANOVA followed by Tukey test; Scale bar = 100 µm, n = 14–16, male and female. Images taken at 40× magnification.

**Figure 6:**
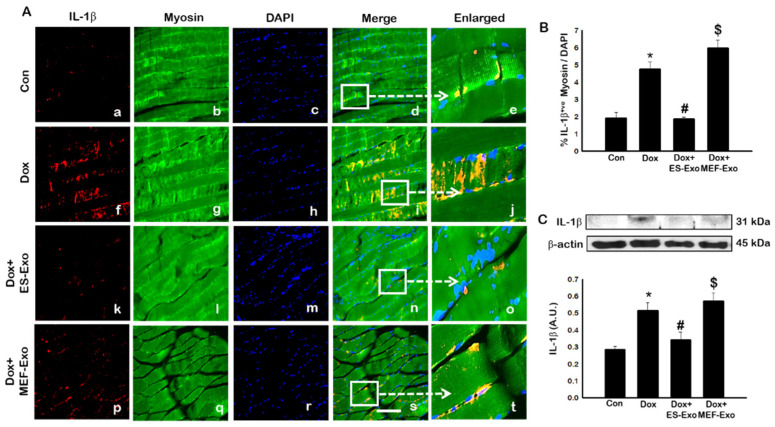
Treatment with ES-Exos reduces IL-1β secretion induced by Dox. (**A**) Representative photomicrographs of soleus muscle sections stained with myosin and pyroptotic marker, IL-1β. Each box shows IL-1β^+^ cells in red (**a**,**f**,**k**,**p**), myocytes in green (**b**,**g**,**l**,**q**), DAPI in blue (**c**,**h**,**m**,**r**), merged images (**d**,**i**,**n**,**s**), and enlarged areas of merged images (**e**,**j**,**o**,**t**). (**B**) Histograms from quantitative analysis of IL-1β^+^ cells over total DAPI. (**C**) Representative blot and densitometric analysis of IL-1β. Error bars = mean ± S.E.M. * *p* < 0.05 vs. control, # *p* < 0.05 vs. Dox, $ *p* = non-significant vs. Dox, one-way ANOVA followed by Tukey test; western blot quantities are represented as A.U. Scale bar = 100 µm, n = 14–16, male and female (**B**) and n = 6–8, male and female (**C**). Images taken at 40× magnification.

**Figure 7:**
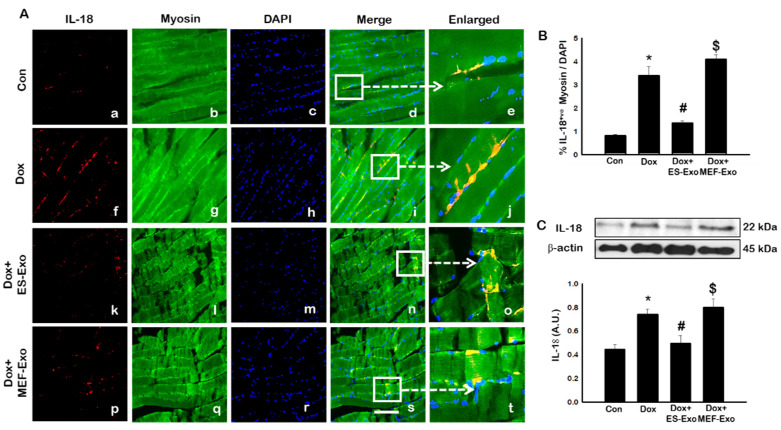
Treatment with ES-Exos reduces IL-18 secretion induced by Dox. (**A**) Representative photomicrographs of soleus muscle sections stained with myosin and pyroptotic marker, IL-18. Each box shows IL-18^+^ cells in red (**a**,**f**,**k**,**p**), myocytes in green (**b**,**g**,**l**,**q**), DAPI in blue (**c**,**h**,**m**,**r**), merged images (**d**,**i**,**n**,**s**), and enlarged areas of merged images (**e**,**j**,**o**,**t**). (**B**) Histograms from quantitative analysis of IL-18^+^ cells over total DAPI. (**C**) Representative blot and densitometric analysis of IL-18. Error bars = mean ± S.E.M. * *p* < 0.05 vs. control, # *p* < 0.05 vs. Dox, $ *p* = non-significant vs. Dox, one-way ANOVA followed by Tukey test; western blot quantities are represented as A.U. Scale bar = 100 µm, n = 14–16, male and female (**B**) and n = 6–8, male and female (**C**). Images taken at 40× magnification.

Inflammasome promotes autocatalytic activation of the cysteine protease caspase-1 and mediates the cleavage of inactive pro-IL-1β and IL-18, among other proteins, into their active forms. Unlike other caspases, caspase-1 is a key modulator of the inflammatory response to tissue injury [38,39,40]. The results show a higher expression of caspase-1 (Figure 5A), IL-1β (Figure 6A), and IL-18 (Figure 7A) after Dox administration (f–j) compared to control (a–e). Also, a significant decrease in the expression of these markers was observed after treatment with ES-Exos (k–o), while MEF-Exos (p–t) had no significant effect on decreasing their expression. The quantitative analysis of caspase-1^+^ (Figure 5B, *p* < 0.05), IL-1β^+^ (Figure 6B, *p* < 0.05) and IL-18^+^ (Figure 7B, *p* < 0.05) cells further confirmed these results as shown by significant increase in their expression in Dox-treated mice and a significant reduction after treatment with ES-Exos (*p* < 0.05).

Similar to IHC staining, Western blot analysis also showed significant increase in IL-1β (Figure 6C, *p* < 0.05) and IL-18 (Figure 7C, *p* < 0.05) levels compared to control mice. Treatment with ES-Exos showed a significant reduction in these pyroptotic markers as compared with Dox-administered mice (*p* < 0.05). Taken together, these results suggest the beneficial effects of ES-Exos in decreasing pyroptosis induced by Dox in the soleus muscle.

### 2.4. ES-Exos Treatment Attenuates Pro-Inflammatory Cytokines Following Dox Administration

**Figure 8:**
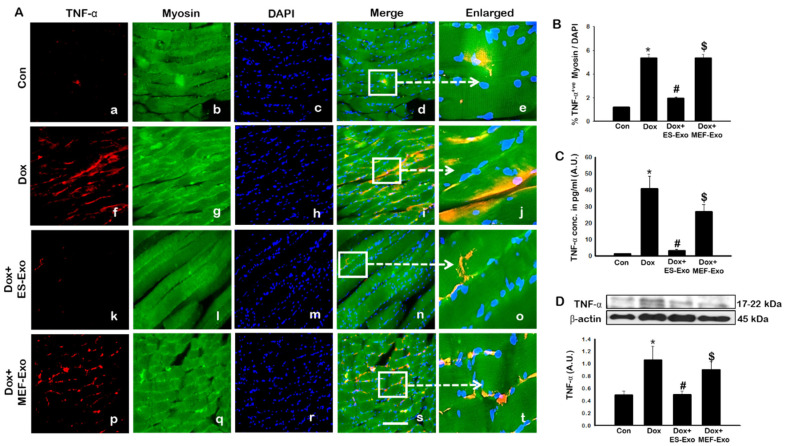
ES-Exos treatment attenuates Dox-induced release of pro-inflammatory cytokine, TNF-α. (**A**) Representative photomicrographs of soleus muscle sections stained with myosin and pro-inflammatory cytokine, TNF-α. Each box shows TNF-α^+^ cells in red (**a**,**f**,**k**,**p**), myocytes in green (**b**,**g**,**l**,**q**), DAPI in blue (**c**,**h**,**m**,**r**), merged images (**d**,**i**,**n**,**s**), and enlarged areas of merged images (**e**,**j**,**o**,**t**). (**B**) Histograms from quantitative analysis of TNF-α^+^ cells over total DAPI. (**C**) Quantification of ELISA analysis for TNF-α. (**D**) Representative blot and densitometric analysis of TNF-α. Error bars = mean ± S.E.M. * *p* < 0.05 vs. control, # *p* < 0.05 vs. Dox, $ *p* = non-significant vs. Dox, one-way ANOVA followed by Tukey test; western blot and ELISA quantities are represented as A.U. Scale bar = 100 µm, n = 14–16, male and female (**B**) and n = 6–8, male and female (**C**,**D**). Images taken at 40× magnification.

**Figure 9:**
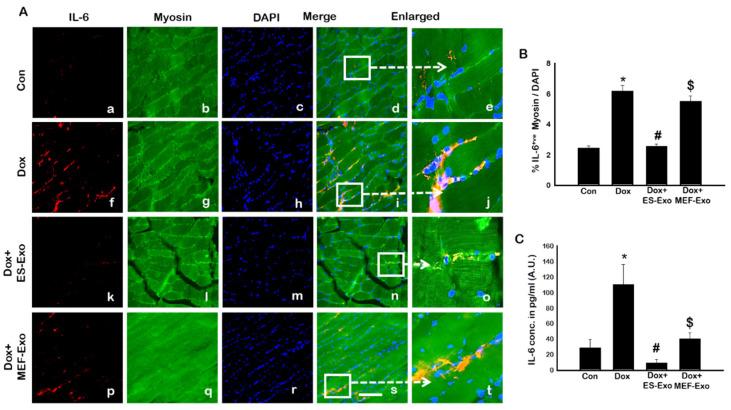
ES-Exos treatment decreases levels of pro-inflammatory cytokine, IL-6, following Dox administration. (**A**) Representative photomicrographs of soleus muscle sections stained with myosin and pro-inflammatory cytokine, IL-6. Each box shows IL-6^+^ cells in red (**a**,**f**,**k**,**p**), myocytes in green (**b**,**g**,**l**,**q**), DAPI in blue (**c**,**h**,**m**,**r**), merged images (**d**,**i**,**n**,**s**), and enlarged areas of merged images (**e**,**j**,**o**,**t**). (**B**) Histograms from quantitative analysis of IL-6^+^ cells over total DAPI. (**C**) Quantification of ELISA analysis for IL-6. Error bars = mean ± S.E.M. * *p* < 0.05 vs. control, # *p* < 0.05 vs. Dox, $ *p* = non-significant vs. Dox, one-way ANOVA followed by Tukey test; ELISA quantities are represented as A.U. Scale bar = 100 µm, n = 14–16, male and female (**B**) and n = 5–7, male and female (**C**). Images taken at 40× magnification.

Since pyroptosis is mediated by inflammation [5,19,22,25], we sought to further investigate the effect of ES-Exos in attenuation of pro-inflammatory cytokines TNF-α and IL-6 following Dox administration in the soleus muscle. Figure 8A and Figure 9A show representative IHC images for TNF-α and IL-6, respectively, which demonstrate increase in the expression of both these pro-inflammatory cytokines in the Dox group (f–j) compared to control group (a–e). Importantly, ES-Exos caused a reduction in TNF-α and IL-6 (k-o), whereas MEF-Exos did not show a difference compared with Dox group (p-t). Quantification showed a significant increase in TNF-α^+^ (Figure 8B, *p* < 0.05) and IL-6^+^ (Figure 9B, *p* < 0.05) cells in the soleus muscle following Dox administration as compared to controls. Additionally, treatment with ES-Exos significantly reduced both TNF-α and IL-6 expressions (*p* < 0.05).

To strengthen and confirm our IHC findings, we performed western blot analysis for TNF-α. Densitometric analysis showed that Dox mice had a significant increase in expression of TNF-α as compared to the control group (Figure 8C, *p* < 0.05), while treatment with ES-Exos significantly reduced its expression compared to Dox (*p* < 0.05). Likewise, treatment with MEF-Exos did not appear to have any significant difference when compared to Dox. These results suggest that ES-Exos have the ability to reduce inflammation in the soleus muscle following Dox administration.

According to the ELISA test for both TNF-α and IL-6, a significant increase was observed in the Dox mice vs. control (Figure 8C and Figure 9C, *p* < 0.05), whereas a significant reduction occurred for the mice treated with ES-Exos compared to Dox alone (*p* < 0.05).

### 2.5. ES-Exos Modulate M1 Macrophages into M2 Macrophages following Dox Treatment

Macrophage polarization plays a crucial role in pyroptosis and inflammation [21,41]. Therefore, we determined macrophage phenotypes and abundance in the soleus tissue by measuring the expression of inducible nitric oxide (iNOS, marker of pro-inflammatory M1 macrophages), CD206 (anti-inflammatory M2 macrophage marker), and arginase-1 (Arg-1, anti-inflammatory M2 macrophage marker) through IHC staining.

**Figure 10:**
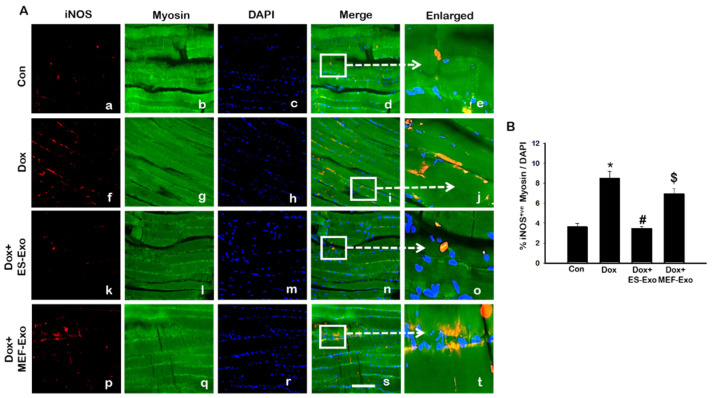
Treatment with ES-Exos reduces pro-inflammatory M1 macrophage abundance following Dox administration. (**A**) Representative photomicrographs of soleus muscle sections stained with myosin and M1 macrophage marker, iNOS. Each box shows iNOS^+^ cells in red (**a**,**f**,**k**,**p**), myocytes in green (**b**,**g**,**l**,**q**), DAPI in blue (**c**,**h**,**m**,**r**), merged images (**d**,**i**,**n**,**s**), and enlarged areas of merged images (**e**,**j**,**o**,**t**). (**B**) Histograms from quantitative analysis of iNOS^+^ cells over total DAPI. Error bars = mean ± S.E.M. * *p* < 0.05 vs. control, # *p* < 0.05 vs. Dox, $ *p* = non-significant vs. Dox, one-way ANOVA followed by Tukey test; Scale bar = 100 µm, n = 14–16, male and female. Images taken at 40× magnification.

Representative images show an increased staining of iNOS (Figure 10A) in the mice administered with Dox (f–j) vs. control (a–e), and a subsequent reduction in expression for ES-Exos-treated mice (k–o). At the same time, treatment with MEF-Exos did not have any effect on iNOS levels, which remained high as compared to ES-Exos treated group (p–t). Quantification of iNOS expression further confirmed these results (Figure 10B, *p* < 0.05). Also, treatment with ES-Exos significantly diminished the levels of iNOS in the soleus muscle (*p* < 0.05), while MEF-Exos had no such effect on the Dox-treated mice. This indicates that treatment with ES-Exos may reduce polarization into pro-inflammatory M1 macrophages.

**Figure 11:**
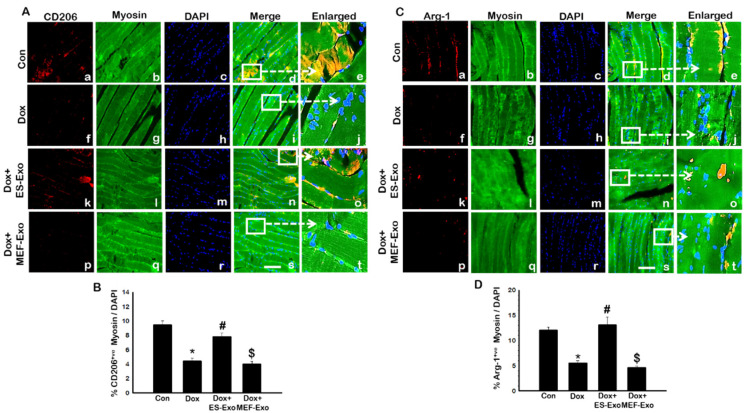
ES-Exos enhances levels of anti-inflammatory M2 macrophage markers, CD206 and Arg-1 in Dox mice. (**A**) Representative photomicrographs of soleus muscle sections stained with myosin and M2 macrophage marker, CD206. Each box shows CD206^+^ cells in red (**a**,**f**,**k**,**p**), myocytes in green (**b**,**g**,**l**,**q**), DAPI in blue (**c**,**h**,**m**,**r**), merged images (**d**,**i**,**n**,**s**), and enlarged areas of merged images (**e**,**j**,**o**,**t**). (**B**) Histograms from quantitative analysis of CD206^+^ cells over total DAPI. (**C**) Representative photomicrographs of soleus muscle sections stained with myosin and M2 macrophage marker, Arg-1. Each box shows Arg-1^+^ cells in red (**a**,**f**,**k**,**p**), myocytes in green (**b**,**g**,**l**,**q**), DAPI in blue (**c**,**h**,**m**,**r**), merged images (**d**,**i**,**n**,**s**), and enlarged areas of merged images (**e**,**j**,**o**,**t**). (**D**) Histograms from quantitative analysis of Arg-1^+^ cells over total DAPI. Error bars = mean ± S.E.M. * *p* < 0.05 vs. control, # *p* < 0.05 vs. Dox, $ *p* = non-significant vs. Dox, one-way ANOVA followed by Tukey test; Scale bar = 100 µm, n = 14–16, male and female. Images taken at 40× magnification.

Contrary to M1 macrophage results, anti-inflammatory M2 macrophage markers CD206 (Figure 11A) and Arg-1 (Figure 11C) showed visible decreased levels in the soleus muscle for Dox mice (f–j) vs. control (a–e). ES-Exos rescued M2 macrophage levels after Dox administration (k–o), while MEF-Exos failed to do so (p–t). Quantitative analysis of CD206^+^ and Arg-1^+^ cells in the soleus muscle showed that they were significantly reduced in Dox mice as compared to control (Figure 11B,D, *p* < 0.05), while treatment with ES-Exos significantly rescued anti-inflammatory M2 macrophage levels (*p* < 0.05). MEF-Exos treatment showed no such significant enhancement of M2 macrophage levels, indicating that ES-Exos may demonstrate anti-inflammatory properties by promoting polarization into M2 macrophages.

### 2.6. ES-Exos Treatment Protects Soleus Muscle Cells from Dox-Induced Atrophy

**Figure 12:**
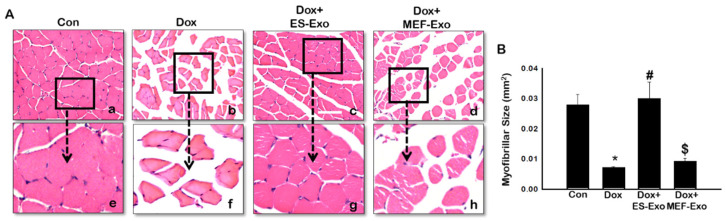
ES-Exos treatment protects the soleus muscle from Dox-induced atrophy. (**A**) Representative images of hematoxylin and eosin (H&E) stained transverse soleus tissue are shown. Each box shows Control (**a**), Dox (**b**), Dox+ES-Exo (**c**), Dox+MEF-Exo (**d**), and enlarged images (**e**–**h**) for each group respectively. (**B**) Quantitative analysis for muscle atrophy. Error bars = mean ± S.E.M. * *p* < 0.05 vs. control, # *p* < 0.05 vs. Dox, $ *p* = non-significant vs. Dox, one-way ANOVA followed by Tukey test; Scale bar = 100 µm, n = 14–16 (male and female). Images taken at 40× magnification.

To evaluate the histological effects of ES-Exos treatment, myofibrillar size on transverse sections of soleus muscle was determined using H&E staining. Representative pictures of the H&E stain (Figure 12A) shows the architecture of the soleus muscle with visible decreased muscle size after Dox administration (b and f) compared to control (a and e). Treatment with ES-Exos enhanced the size of the soleus muscle cell (c and g), while the size remained the same for mice treated with MEF-Exos (d and h) compared to Dox. Our quantitative data showed a significant decrease in myofibrillar size in mice treated with Dox compared to control (Figure 12B, *p* < 0.05), hence exhibiting muscular atrophy. Importantly, ES-Exos treatment group had significantly increased myofibrillar size, displaying protective effects against skeletal muscle atrophy (*p* < 0.05). MEF-Exos failed to display any protective properties in muscle atrophy.

### 2.7. ES-Exos Treatment Decreases Dox-Induced Fibrosis in Soleus Muscle

**Figure 13:**
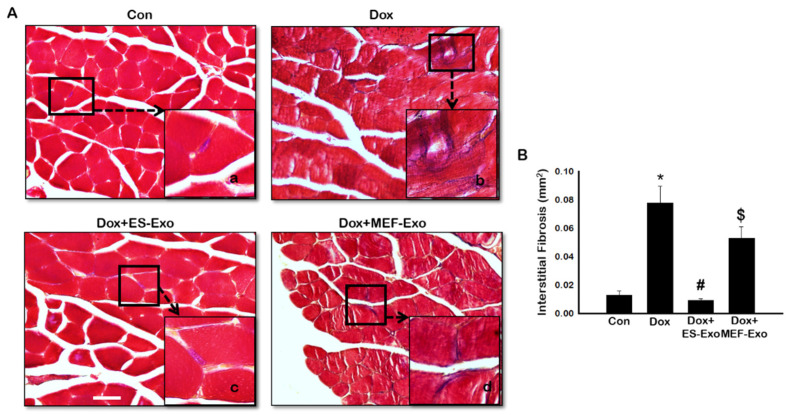
ES-Exos treatment inhibits Dox-induced fibrosis in the soleus muscle. (**A**) Representative Masson’s trichrome images showing interstitial fibrosis in transverse soleus sections. Each box shows Control (**a**), Dox (**b**), Dox+ES-Exo (**c**), and Dox+MEF-Exo (**d**). (**B**) Percentage of interstitial fibrosis quantified over the total muscle cell area. Error bars = mean ± S.E.M. * *p* < 0.05 vs. control, # *p* < 0.05 vs. Dox, $ *p* = non-significant vs. Dox, one-way ANOVA followed by Tukey test; Scale bar = 100 µm, n = 14–16 (male and female). Images taken at 40× magnification.

To evaluate interstitial fibrosis (IF) during skeletal muscle remodeling induced by Dox, and demonstrate the therapeutic effects of ES-Exos against collagen deposition, Masson’s trichrome staining was performed. Representative images show increased IF (Figure 13A) in transverse sections of soleus muscle tissue for Dox mice (Figure 13A(b)) compared to control (Figure 13A(a)). Interestingly, treatment with ES-Exos (Figure 13A(c)) visibly reduced IF while MEF-Exos (Figure 13A(d)) treatment was unable to reduce IF following Dox treatment. Furthermore, quantitative data showed significantly higher fibrosis in Dox-administered mice as compared with control (Figure 13B, *p* < 0.05). Importantly, mice that received ES-Exos as a treatment showed significantly diminished fibrosis within the muscle cells (*p* < 0.05), suggestive of the structural protection provided by treatment with ES-Exos against DIMT. Further, MEF-Exos had no significant effect in reducing fibrosis after Dox treatment.

## 3. Discussion

DIMT is a significant health concern among cancer patients [5,11,12,17]. Patients undergoing chemotherapy through Dox administration have been reported to develop muscle weakness in their lower extremities [42,43]. Cancer patients treated with Dox demonstrated a rapid decline in functional ability and an escalation in fatigue through a walk test [44]. Patients have been reported to show signs of weakness and fatigue 1–5 years after treatment with Dox [45,46,47].

Current therapy against myotoxicity include surgical intervention and antioxidants [2,48,49,50,51]. However, these therapies are limited due to cost, immune rejection and side effects in other organs [51]. In the present investigation, we studied the effect of ES-Exos in attenuating DIMT. The rationale for this approach was that stem cells possess significant therapeutic potential in regeneration and repair of tissues [29,52]. However, human stem cell transplantation therapy is hampered due to their characteristics of teratoma formation (tumorigenesis) [28,29,51]. As such, a cell-free system using secreted exosomes containing the valuable factors of stem cells provide an attractive, viable alternative in avoiding the teratoma formation [5,21,22,32,33].

Apoptosis and necrosis are common forms of cell death implicated as possible causes of skeletal muscle diseases such as Duchenne’s muscular dystrophy, myotonic dystrophy, and diabetes [52,53,54,55]. An increase in cell survival may lead to cancer while accelerated cell death may lead to muscular atrophy, fibrosis and subsequently dysfunction of the muscle. In recent years, inflammation-mediated cell death, known as pyroptosis, has been suggested as a possible mechanism for causing muscle injury [5,25,56]. In the present study, we observed significant reduction of muscle function in Dox-treated animals compared with controls. Such an impairment in muscle function is consistent with increased fatigue and reduced cardiac and muscle function induced by Dox as observed in patients and DIC in animal models [13,21,57]. Furthermore, to investigate whether the reduced muscle function after treatment with Dox is associated with pathological variations, we examined the prevalence of pyroptosis in Dox-treated skeletal muscle. Notably, caspase-1, IL-1β, and IL-18, the well-known markers of pyroptosis, which have been shown to be increased in other disease states such as lung injury, kidney inflammation, cardiomyopathy, and liver damage [21,26,58,59], were increased after Dox treatment. Pyroptosis occurs by inflammation mainly during an infection [19,60,61,62], which is activated by pathogen-associated molecular patterns (PAMPs). Recent studies have also shown that pyroptosis may be activated through sterile inflammation (non-infection) via activation of TLR4 by damage-associated molecular patterns (DAMPs) [5,18,19,22,23]. DAMPs are released cellular debris from either dead or dying cells consisting of ATP, intracellular proteins, S100 proteins, heat-shock proteins, and others [63]. TLR4 activation causes the formation of NLRP3-ASC inflammasome complex formation, which promotes the pyroptosis cascade through proteolytic cleavage of Caspase-1 [24,26,36,64].

In the present study, we observed a significant increase in TLR4 as reported by previous studies [5,18,19,22]. Since stimulated TLR4 receptor activates NLRP3 [22], we examined whether it is upregulated following Dox treatment in the skeletal muscle. Our data showed that increase in TLR4 and NLRP3 was in accord with the published studies showing initiation of TLR4, and the activation of the NLRP3 complex [22,65,66]. The pyroptosis markers were upregulated which are initiated by upregulation of TLR4 and the NLRP3 complex in Dox-induced muscle myopathy. However, the question still remains whether pyroptosis induced in muscle myopathy involves canonical or non-canonical pathway. A canonical pathway involves three main components: (1) a sensor protein, (2) an adaptor, (3) and an effector. So far, we provided evidence of the involvement of TLR4-NLRP3 inflammasome, which are considered as sensor proteins and subsequently cause an increase in caspase-1, called an effector protein, in the pyroptosis pathway [27,58,65,67]. Therefore, we examined the presence of an adaptor protein, ASC, to complete the canonical pathway in Dox-muscle myopathy. Our data showed a significant upregulation of ASC protein, which is in agreement with other published studies in the involvement of pyroptosis mediated through a canonical pathway [27,65,67].

It is widely accepted that pyroptosis is initiated through and further mediates inflammation; therefore, it is imperative to examine inflammation and its source in Dox-induced muscle myopathy. Our data showed a significant increase in pro-inflammatory cytokines TNF-α and IL-6 in Dox-induced muscle myopathy, suggesting the role of inflammation in muscle myopathy. These results are in accordance with other published studies on inflammation in pyroptosis, which state the involvement of TNF-α and IL-6, among others, in propagating and furthering inflammation during pyroptosis [5,19,22,25,58].

Monocyte infiltration and polarization of macrophages play a central role in inflammation [68]. Macrophages undergo activation to either pro-inflammatory M1 or anti-inflammatory M2 phenotype, depending on environmental cues [68]. We have previously shown the involvement of macrophages in inflammation and pyroptosis in cardiac toxicity after Dox exposure [21]. Studies have shown that M1 macrophages, which are pro-inflammatory, arise from TNF-α and simultaneously propagate inflammation through the release of pro-inflammatory cytokines IL-1β and TNF-α [68], thereby contributing to the pyroptosis cycle. Also, interestingly, it is known that macrophages are important for skeletal muscle regeneration through mitogenic and anti-apoptotic activities [69]. It has been reported that monocytes exhibiting inflammatory characteristics may be recruited during skeletal muscle injury, and convert to anti-inflammatory M2 macrophages, stimulating muscle repair and growth [68,69]. Therefore, it is of importance to investigate the source of inflammation and the possible repair of damaged skeletal muscle tissue in the soleus muscle through examination of M1 and M2 macrophages.

Our results showed a significant increase in M1 macrophages as demonstrated by the expression of specific marker, iNOS, for Dox compared to controls. Furthermore, the anti-inflammatory M2 macrophages were significantly reduced following Dox administration in the soleus muscle. These results suggest that inflammation associated with pyroptosis may be correlated with the relative levels of M1 and M2 macrophages in the DIMT model. Similarly, increased infiltration of M1 macrophages have been reported to occur in several other diseases including atherosclerosis, diabetes, and myocardial infarction [41,70,71]. However, further studies are needed to establish the direct link of M1 vs. M2 macrophages associated with the increase of pyroptosis in DIMT.

Drastic change in cell size, such as hypertrophy, and formation of fibrotic tissue are well known to occur following pyroptosis and inflammation in the heart, leading to cardiac dysfunction as shown previously by us [21,32,57]. H&E and Masson’s trichrome staining showed that Dox caused a significant decrease in myofibril size, leading to muscular atrophy. Further, there was a significant increase in interstitial fibrosis within the soleus muscle with Dox administration compared to control. Thus, our results on pyroptosis mediated through NLRP3-ASC inflammasome formation, inflammation, and M1 macrophage levels leading to muscle atrophy and fibrosis may cause muscle atrophy as demonstrated by deterioration of muscle strength following Dox administration.

Weights test, IHC, H&E, and Masson’s trichrome on the soleus muscle were performed which showed that ES-Exos therapy improved muscle function and increased M2 macrophage levels while reducing the pyroptotic cascade, secretion of pro-inflammatory cytokines, M1 macrophages, skeletal muscle atrophy, and fibrosis. These results are consistent with our previous studies on soleus (Sol8) cells [5], cardiomyoblast (H9c2) cells [22] and the heart [21]. IHC and western blot data confirmed a significant decrease in inflammasome formation, pyroptosis, and inflammation after treatment with ES-Exos. Additionally, treatment with MEF-Exos, had no therapeutic effect on the soleus muscle, suggesting that the only factors released from ES cells were critical in quelling pyroptosis, inflammation, and preserving cell structural integrity by reducing muscle atrophy and fibrosis following Dox administration.

The difference in effective therapeutic potential between MEF-Exos and ES-Exos is due to MEF-Exos being derived from fibroblasts, while ES-Exos being derived from stem cells, which are more repair-oriented compared to fibroblasts [5,21,22]. Exosomes contain several proteins, lipids, genetic material (RNA, miRNA, etc.), and growth factors of the parental cell [5,21,22] which may have therapeutic effects against pyroptosis and inflammation. Moreover, we have reported that ES-Exos and MEF-Exos do not show beneficial effects in inhibiting pyroptosis in control conditions; therefore, we do not anticipate that injecting these exosomes in the control animals would reach at any significant beneficial effects [22]. Therefore, we predict that to examine the beneficial effects of ES-Exos, there must be an injury in the model system. Furthermore, using a cytokine array, we recently investigated the composition of ES-Exos and showed a noticeable increase in anti-inflammatory cytokines IL-9, IL-13, and IL-4 as well as lower levels of pro-inflammatory proteins IL-12, TNF-α, TNFR1, and Fas Ligand [22]. These results suggested that the exosomes released from ES cells might be creating an anti-inflammatory microenvironment.

## 4. Materials and Methods

### 4.1. Animal Model and Experimental Design

The University of Central Florida and the Institutional Animal Care and Use Committee (IACUC) approved all animal protocols under protocol number 17–41 on 23 August 2017. C57BL/6J mice (10 ± 2 weeks of age, N = 64 animals) were maintained in a sterile environment and given all appropriate nutrition ad libitum. Four groups of mice underwent the following treatments: Control (saline), Dox, Dox+ES-Exos, and Dox+MEF-Exos (used as negative control), with n = 16 (8 males and 8 females) in each group. Dox was prepared in sterilized distilled water and administered at 4 mg/kg bodyweight (BW) via 3 intraperitoneal (i.p.) injections on alternative days, giving a cumulative dose of 12 mg/kg BW. Control mice received three i.p. injections of 0.9% saline on alternative days. Finally, ES-Exos or MEF-Exos were administered by three i.p. injections on alternative days between Dox treatments with a 50 µg dose, giving a cumulative dose of 150 µg. Mice were sacrificed 14 days (D14) after the last injection of Dox by cervical dislocation after anesthetization with 4% isoflurane via nose cone. Muscle function was performed prior to sacrifice to evaluate the difference in muscle strength between groups. Soleus muscles were harvested and either flash frozen for western blot analysis or kept in 4% paraformaldehyde (PFA, ThermoFisher Scientific, Waltham, MA, USA) for immunohistochemical (IHC) and histological staining.

### 4.2. Cell Culture and Exosome Preparation

Cells lines for ES cells (CGR8, mouse embryonic stem cells) and mouse embryonic fibroblasts (MEF) were purchased from the American Type Culture Collection (ATCC, Manassas, VA, USA). ES cells were cultured in a gelatin-coated tissue culture plate in DMEM containing 15% ES-fetal bovine serum (ES-FBS), β-mercaptoethanol, glutamine, penicillin/streptomycin (P/S), leukemia inhibitory factor (LIF), and sodium pyruvate (NaP). MEF cells were cultured in DMEM containing 10% FBS, NaP, P/S, and glutamine. After 48 h, the cell culture medium was discarded and serum-free knockout DMEM and P/S were added (ThermoFisher Scientific). Exosome (Exos) isolation was performed according to the manufacturer’s protocol of the Exoquick TC exosome isolation kit (SBI, Palo Alto, CA, USA). The isolated Exos were characterized by performing western blot on exosome markers HSP-70 and CD63, as we have recently shown [22].

### 4.3. Muscle Function Analysis

Muscle function was performed as previously reported by Deacon [34]. Briefly, mice were allowed to carry different weights ranging from 15–65 g, which were attached to a mesh wire for 3 s. If the weight hold was longer than 3 s, the mouse was allowed to progress to the next weight. If the hold was less than 3 s, then the mouse was given three attempts for that particular weight with resting periods of 5 min between each weight and trial.

Analysis of muscle function was calculated in two ways; weight by time (WT) and trial by time (TT). For WT, the weight carried by the mouse was multiplied by time (i.e., 55 g × 3 s = 165 pts) of hold and expressed in arbitrary units (A.U.). For TT, the trial number was multiplied by the time the weight was held and expressed in arbitrary units (A.U.). The bar graph was plotted using SigmaPlot software.

### 4.4. Immunohistochemistry Staining

After 24–48 h, PFA-stored tissues were processed overnight through Leica TP 1020 (Leica Biosystems, Buffalo Grove, IL, USA), embedded in paraffin, then sectioned at a thickness of 5 μm. Soleus tissue sections were placed on microscope slides and double IHC staining was accomplished as previously shown [11,21,35,36]. Sections were deparaffinized, rehydrated, and then blocked for 1 h at room temperature (RT) with 10% normal goat serum or normal donkey serum (Vector Laboratories, Burlingame, CA, USA) preceding an overnight incubation at 4 °C with primary antibody for Myosin (1:30 dilution; Cat# M7523–1 ML; Sigma Aldrich, St. Louis, MO, USA). Tissues were then incubated with secondary antibody, Alexa 488 goat anti-rabbit (Cat# A11008; ThermoFisher Scientific, Waltham, MA, USA) at a 1:50 dilution. Next, tissues were blocked a second time with normal goat or donkey serum followed by an overnight incubation at 4 °C with primary antibodies for inflammasome, pyroptotic, and macrophage markers, all at 1:50 dilutions (except for IL-6 at 1:400). These include Toll-like receptor 4 (TLR4, Cat# ab13556; Abcam, Cambridge, MA, USA), Nucleotide-binding oligomerization domain (NOD) leucine-rich repeats and pyrin domain containing protein 3 (NLRP3, Cat# ab214185; Abcam), and Apoptosis-associated speck-like protein containing a caspase recruitment domain (ASC, Cat# SAB4501315-100 UG; Sigma Aldrich) for inflammasome formation. Primary antibodies used for markers for pyroptosis were caspase-1 (Cat# ab138483; Abcam), Interleukin (IL)-1β (Cat# ab2105; Abcam), and IL-18 (Cat# ab207323; Abcam). Tumor necrosis factor-α (TNF-α, Cat# ab6671; Abcam) and IL-6 (Cat# ab6672; Abcam) primary antibodies were used for pro-inflammatory cytokines. Primary antibody for inducible Nitric Oxide Synthase (iNOS, Cat# ab15323; Abcam) was used to detect M1 macrophage levels. Cluster of differentiation 206 (CD206, Cat# ab64693; Abcam) and arginase-1 (Arg-1, Cat# SC18351; Sigma Aldrich) primary antibodies were used to detect M2 macrophage levels.

Following incubation with primary antibodies for the inflammasome, pyroptosis, and macrophage markers, sections were incubated with secondary antibody, Alexa 568 goat anti-rabbit for all markers or Alexa 594 donkey anti-goat for Arg-1 (1:50 dilution, Cat# A11011; ThermoFisher Scientific). Finally, slides were covered with mounting medium containing 4,6-diamidino-2-phenylindole (DAPI, Vector Laboratories, Burlingame, CA, USA) and cover slips were placed on the slides. Pictures were taken using Keyence BZ-X810 (Keyence, Itasca, IL, USA) microscope. Quantitative analysis was performed on 4–5 pictures per soleus tissue section to determine the percentage of pyroptotic cell death, inflammatory cytokine secretion and macrophage infiltration by the formula [(total cells^+^/total DAPI) × 100]. Quantification was performed using NIH ImageJ software.

### 4.5. Western Blot

Western blot was completed as previously described by us [5,21,22]. In brief, the soleus muscle was homogenized using radioimmunoprecipictation assay cell lysis buffer and the supernatant was used. Using the Bio-Rad protein assay (Bio-Rad, Hercules, CA, USA), protein concentration was estimated and 50 µg of protein was run onto a 10% or 15% SDS-PAGE gel (150 V for 1 h). Using a semi-dry transfer machine (Bio-Rad), gels were transblotted onto polyvinylidene difluoride membranes (Bio-Rad) and were subsequently blocked with 5% skim milk. Next, the membranes were incubates with primary antibodies overnight at 4 °C for inflammasome markers including TLR4 (1:1000, Cat# ab13556, 90 kDa; Abcam) and NLRP3 (1:1000, Cat# ab214185, 90 kDa; Abcam). Pyroptosis markers were detected using primary antibodies for IL-1β (1:1000, Cat# ab2105, 31 kDa; Abcam), and IL-18 (1:1000, Cat# ab7149, 22 kDa; Abcam). Inflammation was detected by primary antibody for TNF-α at a 1:500 dilution (Cat# ab6671, 17–22 kDa; Abcam). Finally, primary antibody for β-actin was used as a loading control at a 1:1000 dilution (Cat# ab16039, 45 kDa; Cell Signaling Technology, Danvers, MA, USA). Membranes were exposed to enhanced chemiluminescence substrate (ThermoFisher Scientific). Densitometric analysis was performed using NIH ImageJ software.

### 4.6. Enzyme-Linked Immunoassay (ELISA)

ELISA was performed using 50 µL of serum sample according to the manufacturer’s protocol for the mouse TNF-α and IL-6 ELISA kits (Cat# MTA00B for TNF-α and Cat# M6000B for IL-6, R&D Systems, Minneapolis, MN, USA). Using a Bio-Rad plate reader (Bio-Rad) at 450 nm, absorbance data was obtained and a standard curve was used for quantification.

### 4.7. Histological Staining

#### 4.7.1. Hematoxylin and Eosin (H&E)

The histology was performed to determine myofibrillar size and atrophy, as described previously [21]. Transverse soleus sections were stained with H&E (ThermoFisher Scientific). ImageJ was used to determine myocyte size (mm^2^) at 20× magnification and representative images were taken at 40× magnification.

#### 4.7.2. Masson’s Trichrome

Masson’s Trichrome staining was performed to determine fibrosis in the soleus muscle as previously published by us [21]. Interstitial fibrosis was quantified by measuring the total fibrotic area per mm^2^ on 20× magnified images (3–4 images/tissue) using ImageJ software. Representative images were taken at 40× magnification.

### 4.8. Statistical Analysis

One-way analysis of variance (ANOVA) and Tukey test were used to analyze the data. All data is expressed as a mean ± standard error of mean (SEM). The *p*-value < 0.05 denotes statistical significance. Bar graphs were plotted using SigmaPlot software.

## 5. Conclusions

We report for the first time that Dox-induced muscle myopathy is mediated by pyroptosis through a canonical pathway. We also report novel findings in cancer drug-induced muscle myopathy involving inflammation and upregulation of pro-inflammatory M1 macrophages. In addition, these results suggest that ES-Exos are beneficial carriers of therapeutic agents in suppressing inflammation as well as improving muscle function. We propose that the inhibition of pyroptosis by ES-Exos may have far-reaching therapeutic implications in rescuing tissue injury against inflammation-induced cell death under various disease states such as ischemia/reperfusion and cancer drug toxicity in multiple organs. Despite excellent therapeutic efficacy of ES-Exos against DIMT, one limitation of this study is that it is not clear which specific factor(s) may be responsible for such an impressive effect. There is good possibility that exosomes contain many more classes of bioactive molecules than previously recognized and no single RNA species can account for all the benefits of exosomes. It is probably the totality of exosomal contents which may be required for full manifestation of bioactivity. Thus, future studies are required to fully characterize the contribution(s) of ES-Exos-derived endogenous factors in their protective effects.

## References

[1] Gewirtz D.A. (1999). A critical evaluation of the mechanisms of action proposed for the antitumor effects of the anthracycline antibiotics adriamycin and daunorubicin. Biochem. Pharmacol..

[2] Qin X.J., He W., Hai C.X., Liang X., Liu R. (2008). Protection of multiple antioxidants Chinese herbal medicine on the oxidative stress induced by adriamycin chemotherapy. J. Appl. Toxicol..

[3] Tacar O., Sriamornsak P., Dass C.R. (2013). Doxorubicin: An update on anticancer molecular action, toxicity and novel drug delivery systems. J. Pharm. Pharmacol..

[4] Turakhia S., Venkatakrishnan C.D., Dunsmore K., Wong H., Kuppusamy P., Zweier J.L., Ilangovan G. (2007). Doxorubicin-induced cardiotoxicity: Direct correlation of cardiac fibroblast and H9c2 cell survival and aconitase activity with heat shock protein 27. Am. J. Physiol. Heart Circ. Physiol..

[5] Dargani Z.T., Singla R., Johnson T., Kukreja R., Singla D.K. (2018). Exosomes derived from embryonic stem cells inhibit doxorubicin and inflammation-induced pyroptosis in muscle cells. Can. J. Physiol. Pharmacol..

[6] Hudis C.A., Schmitz N. (2004). Dose-dense chemotherapy in breast cancer and lymphoma. Semin. Oncol..

[7] You J., Gao F., Tang H., Peng F., Jia L., Huang K., Chow K., Zhao J., Liu H., Lin Y. (2019). A medicinal and edible formula YH0618 ameliorates the toxicity induced by Doxorubicin via regulating the expression of Bax/Bcl-2 and FOXO4. J. Cancer.

[8] Cosgriff T.M. (1980). Doxorubicin and ventricular arrhythmia. Ann. Intern. Med..

[9] Kilickap S., Barista I., Akgul E., Aytemir K., Aksoy S., Tekuzman G. (2007). Early and late arrhythmogenic effects of doxorubicin. South Med. J..

[10] Powis G., Kooistra K.L. (1987). Doxorubicin-induced hair loss in the Angora rabbit: A study of treatments to protect against the hair loss. Cancer Chemother. Pharmacol..

[11] Merino H., Singla D.K. (2018). Secreted Frizzled-Related Protein-2 Inhibits Doxorubicin-Induced Apoptosis Mediated through the Akt-mTOR Pathway in Soleus Muscle. Oxid. Med. Cell Longev..

[12] Hiensch A.E., Bolam K.A., Mijwel S., Jeneson J.A.L., Huitema A.D.R., Kranenburg O., van der Wall E., Rundqvist H., Wengstrom Y., May A.M. (2019). Doxorubicin-induced skeletal muscle atrophy: Elucidating the underlying molecular pathways. Acta. Physiol. (Oxf.).

[13] Bonifati D.M., Ori C., Rossi C.R., Caira S., Fanin M., Angelini C. (2000). Neuromuscular damage after hyperthermic isolated limb perfusion in patients with melanoma or sarcoma treated with chemotherapeutic agents. Cancer Chemother. Pharmacol..

[14] Gilliam L.A., St Clair D.K. (2011). Chemotherapy-induced weakness and fatigue in skeletal muscle: The role of oxidative stress. Antioxid. Redox. Signal.

[15] McLoon L.K., Wirtschafter J.D., Cameron J.D. (1993). Muscle loss from doxorubicin injections into the eyelids of a patient with blepharospasm. Am. J. Ophthalmol..

[16] Valiyil R., Christopher-Stine L. (2010). Drug-related myopathies of which the clinician should be aware. Curr. Rheumatol. Rep..

[17] Smuder A.J., Kavazis A.N., Min K., Powers S.K. (2011). Exercise protects against doxorubicin-induced oxidative stress and proteolysis in skeletal muscle. J. Appl. Physiol..

[18] Fink S.L., Cookson B.T. (2005). Apoptosis, pyroptosis, and necrosis: Mechanistic description of dead and dying eukaryotic cells. Infect Immun..

[19] Man S.M., Karki R., Kanneganti T.D. (2017). Molecular mechanisms and functions of pyroptosis, inflammatory caspases and inflammasomes in infectious diseases. Immunol. Rev..

[20] Li P., Zhong X., Li J., Liu H., Ma X., He R., Zhao Y. (2018). MicroRNA-30c-5p inhibits NLRP3 inflammasome-mediated endothelial cell pyroptosis through FOXO3 down-regulation in atherosclerosis. Biochem. Biophys. Res. Commun..

[21] Singla D.K., Johnson T.A., Dargani Z.T. (2019). Exosome Treatment Enhances Anti-Inflammatory M2 Macrophages and Reduces Inflammation-Induced Pyroptosis in Doxorubicin-Induced Cardiomyopathy. Cells.

[22] Dargani Z.T., Singla D.K. (2019). Embryonic stem cell-derived exosomes inhibit doxorubicin-induced TLR4-NLRP3-mediated cell death-pyroptosis. Am. J. Physiol. Heart Circ. Physiol..

[23] Yu Z.W., Zhang J., Li X., Wang Y., Fu Y.H., Gao X.Y. (2020). A new research hot spot: The role of NLRP3 inflammasome activation, a key step in pyroptosis, in diabetes and diabetic complications. Life Sci..

[24] Fang R., Uchiyama R., Sakai S., Hara H., Tsutsui H., Suda T., Mitsuyama M., Kawamura I., Tsuchiya K. (2019). ASC and NLRP3 maintain innate immune homeostasis in the airway through an inflammasome-independent mechanism. Mucosal. Immunol..

[25] Haldar S., Dru C., Choudhury D., Mishra R., Fernandez A., Biondi S., Liu Z., Shimada K., Arditi M., Bhowmick N.A. (2015). Inflammation and pyroptosis mediate muscle expansion in an interleukin-1beta (IL-1beta)-dependent manner. J. Biol. Chem..

[26] Hou L., Yang Z., Wang Z., Zhang X., Zhao Y., Yang H., Zheng B., Tian W., Wang S., He Z. (2018). NLRP3/ASC-mediated alveolar macrophage pyroptosis enhances HMGB1 secretion in acute lung injury induced by cardiopulmonary bypass. Lab Investig..

[27] Gong W., Shi Y., Ren J. (2019). Research progresses of molecular mechanism of pyroptosis and its related diseases. Immunobiology.

[28] Blum B., Benvenisty N. (2008). The tumorigenicity of human embryonic stem cells. Adv. Cancer Res..

[29] Faiella W., Atoui R. (2016). Therapeutic use of stem cells for cardiovascular disease. Clin. Transl. Med..

[30] Gordeeva O., Khaydukov S. (2017). Tumorigenic and Differentiation Potentials of Embryonic Stem Cells Depend on TGFbeta Family Signaling: Lessons from Teratocarcinoma Cells Stimulated to Differentiate with Retinoic Acid. Stem. Cells Int..

[31] Hentze H., Soong P.L., Wang S.T., Phillips B.W., Putti T.C., Dunn N.R. (2009). Teratoma formation by human embryonic stem cells: Evaluation of essential parameters for future safety studies. Stem. Cell Res..

[32] Deng S., Zhou X., Ge Z., Song Y., Wang H., Liu X., Zhang D. (2019). Exosomes from adipose-derived mesenchymal stem cells ameliorate cardiac damage after myocardial infarction by activating S1P/SK1/S1PR1 signaling and promoting macrophage M2 polarization. Int. J. Biochem. Cell Biol..

[33] Sahoo S., Losordo D.W. (2014). Exosomes and cardiac repair after myocardial infarction. Circ. Res..

[34] Deacon R.M. (2013). Measuring the strength of mice. J. Vis. Exp..

[35] Ghonime M.G., Shamaa O.R., Eldomany R.A., Gavrilin M.A., Wewers M.D. (2012). Tyrosine phosphatase inhibition induces an ASC-dependent pyroptosis. Biochem. Biophys. Res. Commun..

[36] Li J.Y., Wang Y.Y., Shao T., Fan D.D., Lin A.F., Xiang L.X., Shao J.Z. (2020). The zebrafish NLRP3 inflammasome has functional roles in ASC-dependent interleukin-1beta maturation and gasdermin E-mediated pyroptosis. J. Biol. Chem..

[37] Mariathasan S., Newton K., Monack D.M., Vucic D., French D.M., Lee W.P., Roose-Girma M., Erickson S., Dixit V.M. (2004). Differential activation of the inflammasome by caspase-1 adaptors ASC and Ipaf. Nature.

[38] Cassel S.L., Sutterwala F.S. (2010). Sterile inflammatory responses mediated by the NLRP3 inflammasome. Eur. J. Immunol..

[39] Hornung V., Latz E. (2010). Critical functions of priming and lysosomal damage for NLRP3 activation. Eur. J. Immunol..

[40] Tschopp J., Schroder K. (2010). NLRP3 inflammasome activation: The convergence of multiple signalling pathways on ROS production?. Nat. Rev. Immunol..

[41] Singla D.K., Singla R., Wang J. (2016). BMP-7 Treatment Increases M2 Macrophage Differentiation and Reduces Inflammation and Plaque Formation in Apo E-/- Mice. PLoS ONE.

[42] Harada Y., Kato S., Komiya H., Shirota T., Mukai K., Hayashi T. (2004). Primary omental gamma/delta T-cell lymphoma involving the central nervous system. Leuk. Lymphoma.

[43] Jhamb R., Gupta N., Garg S., Kumar S., Gulati S., Mishra D., Beniwal P. (2007). Diffuse lymphomatous infiltration of kidney presenting as renal tubular acidosis and hypokalemic paralysis: Case report. Croat Med. J..

[44] Schwartz A.L., Winters-Stone K., Gallucci B. (2007). Exercise effects on bone mineral density in women with breast cancer receiving adjuvant chemotherapy. Oncol. Nurs. Forum..

[45] Elbl L., Vasova I., Tomaskova I., Jedlicka F., Kral Z., Navratil M., Smardova L., Wagnerova B., Vorlicek J. (2006). Cardiopulmonary exercise testing in the evaluation of functional capacity after treatment of lymphomas in adults. Leuk. Lymphoma.

[46] Turner-Gomes S.O., Lands L.C., Halton J., Hanning R.M., Heigenhauser G.J., Pai M., Barr R. (1996). Cardiorespiratory status after treatment for acute lymphoblastic leukemia. Med. Pediatr. Oncol..

[47] Villani F., Busia A., Villani M., Laffranchi A., Viviani S., Bonfante V. (2009). Cardiopulmonary response to exercise in patients with different degrees of lung toxicity after radio-chemotherapy for Hodgkin’s disease. Anticancer Res..

[48] Martinez P.F., Bonomo C., Guizoni D.M., Junior S.A., Damatto R.L., Cezar M.D., Lima A.R., Pagan L.U., Seiva F.R., Bueno R.T. (2016). Modulation of MAPK and NF-954;B Signaling Pathways by Antioxidant Therapy in Skeletal Muscle of Heart Failure Rats. Cell Physiol. Biochem..

[49] Goetsch M.F. (2007). Surgery combined with muscle therapy for dyspareunia from vulvar vestibulitis: An observational study. J. Reprod. Med..

[50] Sung V.W., Borello-France D., Newman D.K., Richter H.E., Lukacz E.S., Moalli P., Weidner A.C., Smith A.L., Dunivan G., Ridgeway B. (2019). Effect of Behavioral and Pelvic Floor Muscle Therapy Combined with Surgery vs. Surgery Alone on Incontinence Symptoms Among Women with Mixed Urinary Incontinence: The ESTEEM Randomized Clinical Trial. JAMA.

[51] Swijnenburg R.J., Tanaka M., Vogel H., Baker J., Kofidis T., Gunawan F., Lebl D.R., Caffarelli A.D., de Bruin J.L., Fedoseyeva E.V. (2005). Embryonic stem cell immunogenicity increases upon differentiation after transplantation into ischemic myocardium. Circulation.

[52] Yan B., Singla D.K. (2013). Transplanted induced pluripotent stem cells mitigate oxidative stress and improve cardiac function through the Akt cell survival pathway in diabetic cardiomyopathy. Mol. Pharm..

[53] Bhagavati S., Leung B., Shafiq S.A., Ghatpande A. (1997). Myotonic dystrophy: Decreased levels of myotonin protein kinase (Mt-PK) leads to apoptosis in muscle cells. Exp. Neurol..

[54] Loro E., Rinaldi F., Malena A., Masiero E., Novelli G., Angelini C., Romeo V., Sandri M., Botta A., Vergani L. (2010). Normal myogenesis and increased apoptosis in myotonic dystrophy type-1 muscle cells. Cell Death Differ..

[55] Migheli A., Mongini T., Doriguzzi C., Chiado-Piat L., Piva R., Ugo I., Palmucci L. (1997). Muscle apoptosis in humans occurs in normal and denervated muscle, but not in myotonic dystrophy, dystrophinopathies or inflammatory disease. Neurogenetics.

[56] Yu L.M., Zhang W.H., Han X.X., Li Y.Y., Lu Y., Pan J., Mao J.Q., Zhu L.Y., Deng J.J., Huang W. (2019). Hypoxia-Induced ROS Contribute to Myoblast Pyroptosis during Obstructive Sleep Apnea via the NF-kappaB/HIF-1alpha Signaling Pathway. Oxid. Med. Cell Longev..

[57] Singla D.K., Ahmed A., Singla R., Yan B. (2012). Embryonic stem cells improve cardiac function in Doxorubicin-induced cardiomyopathy mediated through multiple mechanisms. Cell Transpl..

[58] Lorenz G., Darisipudi M.N., Anders H.J. (2014). Canonical and non-canonical effects of the NLRP3 inflammasome in kidney inflammation and fibrosis. Nephrol. Dial. Transpl..

[59] Zhang M., Zhu X., Tong H., Lou A., Li Y., Li Y., Su L., Li X. (2019). AVE 0991 Attenuates Pyroptosis and Liver Damage after Heatstroke by Inhibiting the ROS-NLRP3 Inflammatory Signalling Pathway. Biomed. Res. Int..

[60] Cheung K.T., Sze D.M., Chan K.H., Leung P.H. (2018). Involvement of caspase-4 in IL-1 beta production and pyroptosis in human macrophages during dengue virus infection. Immunobiology.

[61] Liu X., Lieberman J. (2017). A Mechanistic Understanding of Pyroptosis: The Fiery Death Triggered by Invasive Infection. Adv. Immunol..

[62] Ryu J.C., Kim M.J., Kwon Y., Oh J.H., Yoon S.S., Shin S.J., Yoon J.H., Ryu J.H. (2017). Neutrophil pyroptosis mediates pathology of P. aeruginosa lung infection in the absence of the NADPH oxidase NOX2. Mucosal. Immunol..

[63] Roh J.S., Sohn D.H. (2018). Damage-Associated Molecular Patterns in Inflammatory Diseases. Immune Netw..

[64] Li D., Ren W., Jiang Z., Zhu L. (2018). Regulation of the NLRP3 inflammasome and macrophage pyroptosis by the p38 MAPK signaling pathway in a mouse model of acute lung injury. Mol. Med. Rep..

[65] Pellegrini C., Antonioli L., Lopez-Castejon G., Blandizzi C., Fornai M. (2017). Canonical and Non-Canonical Activation of NLRP3 Inflammasome at the Crossroad between Immune Tolerance and Intestinal Inflammation. Front. Immunol..

[66] Toldo S., Mezzaroma E., McGeough M.D., Pena C.A., Marchetti C., Sonnino C., Van Tassell B.W., Salloum F.N., Voelkel N.F., Hoffman H.M. (2015). Independent roles of the priming and the triggering of the NLRP3 inflammasome in the heart. Cardiovasc. Res..

[67] Kayagaki N., Warming S., Lamkanfi M., Vande Walle L., Louie S., Dong J., Newton K., Qu Y., Liu J., Heldens S. (2011). Non-canonical inflammasome activation targets caspase-11. Nature.

[68] Kharraz Y., Guerra J., Mann C.J., Serrano A.L., Munoz-Canoves P. (2013). Macrophage plasticity and the role of inflammation in skeletal muscle repair. Mediat. Inflamm..

[69] Arnold L., Henry A., Poron F., Baba-Amer Y., van Rooijen N., Plonquet A., Gherardi R.K., Chazaud B. (2007). Inflammatory monocytes recruited after skeletal muscle injury switch into antiinflammatory macrophages to support myogenesis. J. Exp. Med..

[70] Shoulders H., Garner K.H., Singla D.K. (2019). Macrophage depletion by clodronate attenuates bone morphogenetic protein-7 induced M2 macrophage differentiation and improved systolic blood velocity in atherosclerosis. Transl. Res..

[71] Singla D.K., Singla R.D., Abdelli L.S., Glass C. (2015). Fibroblast growth factor-9 enhances M2 macrophage differentiation and attenuates adverse cardiac remodeling in the infarcted diabetic heart. PLoS ONE.

